# Facial Nerve Abnormalities in Congenital Middle Ear Malformations With Comments on Preoperative Detectability: A Report of Two Cases

**DOI:** 10.7759/cureus.81488

**Published:** 2025-03-31

**Authors:** Chieko Yokota, Yuki Koda, Yasuyuki Kajimoto, Taro Shimono, Kishiko Sunami

**Affiliations:** 1 Otolaryngology - Head and Neck Surgery, Osaka Metropolitan University, Osaka, JPN; 2 Radiology, Osaka Metropolitan University, Osaka, JPN

**Keywords:** facial nerve abnormalities, high-resolution computed tomography, middle ear malformation, stapes ankylosis, tees

## Abstract

Facial nerve abnormalities and congenital middle ear malformations originate in similar developmental stages and are often concomitant. Preoperative recognition of such a condition is desirable to prevent any damage, but it is often difficult. Here, we report two cases of middle ear malformations associated with facial nerve abnormalities, describe the computed tomography (CT) appearance, and discuss its preoperative detectability.

Case 1 was a 40-year-old female with a facial nerve abnormality associated with stapes ankylosis. She underwent stapes surgery. The facial nerve was hanging over the footplate of the stapes. Case 2 was a 32-year-old female with a facial nerve abnormality associated with stapes ankylosis. She underwent an exploratory tympanotomy. The facial nerve was branched. The branches emerged from the incudo-malleolar joint, ran between the chorda tympani nerve and the incus, and ran into the temporal bone inside the canaliculus chorda tympani. For both cases, the facial nerve branches were detectable with high-resolution CT (HRCT) in the mastoid segment and were confirmed during surgery. For Case 2, the facial nerve was also visible with HRCT as soft tissue shadows in the tympanic portion on the lateral side of the stapes.

These two cases highlighted the critical role of preoperative CT imaging in detecting subtle features of facial nerve anomalies, such as nerve branching in the mastoid segment or soft tissue shadows around ossicles.

## Introduction

Facial nerve abnormalities are very troublesome conditions in middle ear surgery, which can result in facial nerve palsy if overlooked. Facial nerve abnormalities have been reported to occur in approximately 24% to 32% of cases of middle ear malformation [1,2].

The superstructure of the stapes, the tympanic side of the footplate, and the facial nerve originate from the second branchial arch; hence, they share an intimately related developmental history. The Reichert's cartilage, which serves as the precursor to the stapes, first appears in the early seventh week, at which point the tympanic portion of the facial nerve is already formed [3]. If external forces are applied, there is a possibility that the nerve may shift anteriorly before the stapes has fully developed [1,4]. Consequently, in cases of stapes malformation, one must consider the potential for anomalies in the course of the facial nerve [3].

Facial nerve abnormalities have been reported in malformations of the inner, middle, and outer ear [5-7]. In the labyrinthine segment, the facial nerve may be displaced antero-superiorly due to developmental abnormalities of the inner ear [5]. In the tympanic segment, the downward displacement of the facial nerve has been reported, including cases where it is located above or covers the oval window and crosses the promontory [5]. In reports of middle ear malformation, the most common type of associated facial nerve abnormality is overhanging over the oval window. In addition, bifurcation and transverse over the promontory have been reported [1]. In the mastoid segment, the facial nerve tends to shift anteriorly and laterally, shortening the distance to the external auditory canal. This displacement is often observed in association with outer ear malformations [5,7,8]. Along with displacement of the mastoid portion of the facial nerve, the second genu of the facial nerve may also be displaced anteriorly and laterally, which, in some cases, can make it difficult to approach the tympanic cavity during surgery.

High-resolution computed tomography (HRCT) provides clear images of the temporal bone, stapes, ossicular chain, and tensor tympani muscle for middle ear surgeons to prepare for safe surgery [9,10]. In the depiction of the facial nerve within the temporal bone, CT is superior to magnetic resonance imaging (MRI) [11]. As the image clarity of the facial nerve abnormalities may be partially limited, radiological evaluation should integrate information from various parts. Here, we report two cases of middle ear malformations associated with facial nerve abnormalities, describe the CT appearance, and discuss its preoperative detectability. The case reports in this paper were conducted with the consent of the patients.

## Case presentation

Case 1

**Figure 1:**
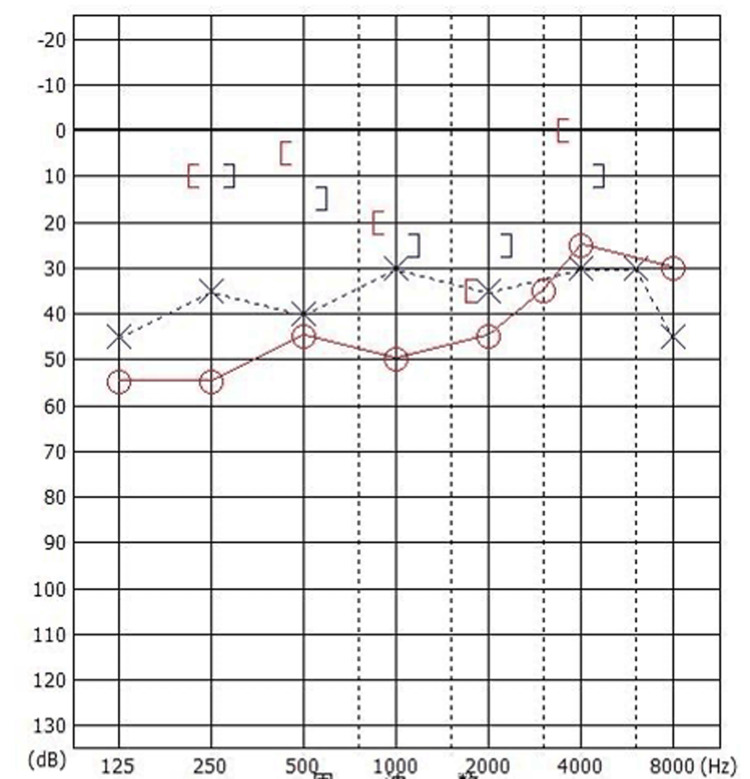
Preoperative pure tone audiogram showing mixed hearing loss in both ears (Case 1). The average hearing level (500 Hz, 1 kHz, and 2 kHz) was 46.7 dB for the right ear (circle) and 35.0 dB for the left ear (cross).

A 40-year-old female visited our hospital with bilateral hearing impairment from childhood. She had a history of left ear surgery in her childhood; however, the details of the surgery were unclear. The external ear canals were normal, and the tympanic membrane looked almost normal except for small calcification lesions. Pure-tone audiometry showed mixed hearing loss in both ears. Her average hearing levels (500 Hz, 1 kHz, and 2 kHz) were 46.7 dB and 35.0 dB in the right and left ear, and the stapedial reflex was absent in both ears (Figure 1).

Radiological Findings

**Figure 2:**
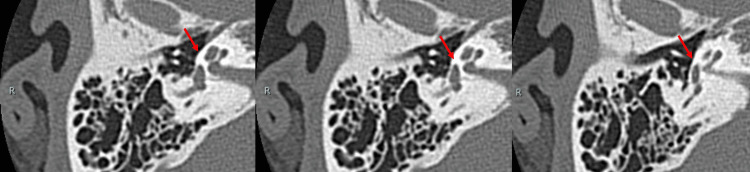
Axial HRCT images of the right temporal bone (Case 1) showing no sign of hypodense demineralized plaques in the region of the fissure ante fenestra (arrows). Three consecutive sections shown are 0.5 mm thick. HRCT, High-resolution computed tomography

**Figure 3:**
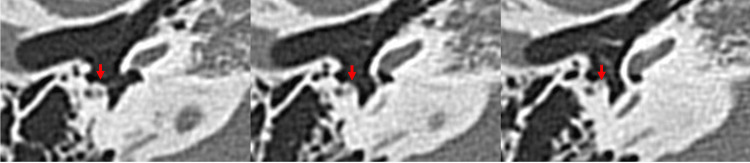
Axial HRCT images of the right temporal bone (Case 1) showing a hypoplastic pyramidal eminence (arrows). Three consecutive sections shown are 0.5 mm thick. HRCT, High-resolution computed tomography

**Figure 4:**
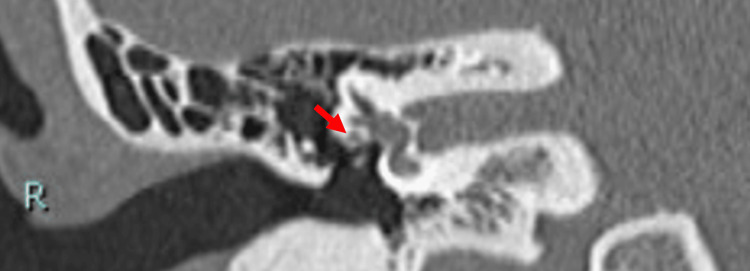
Coronal HRCT images of the right temporal bone showing the normal position of facial nerve (arrow) below the lateral semicircular canal (Case 1). HRCT, High-resolution computed tomography

**Figure 5:**
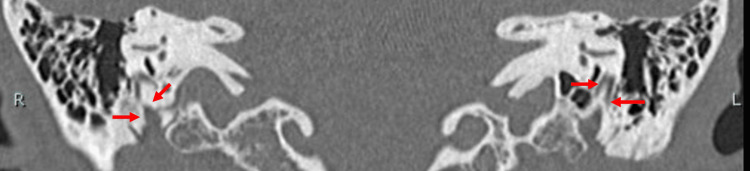
Coronal HRCT image of the temporal bone showing the mastoid segment of facial nerve appearing as an obscured non-single fiber (arrows) on the right (R) side and two separate fibers (arrows) on the left (L) side (Case 1). HRCT, High-resolution computed tomography

A HRCT scan of the temporal bone (120 kVp, high-resolution matrix (512 × 512), section thickness 0.5 mm, and Field of View, or FOV 15-20 cm) showed no hypodense demineralized plaques in the region of the fissure ante fenestra (Figure 2). In the case of otosclerosis, the typical CT finding of 'hypodense demineralized plaques' of the lateral wall of the cochlea is expected but may not be absent. Therefore, not only otosclerosis, but also congenital stapes ankylosis, needed to be considered. There were no ossicular abnormalities. The pyramidal prominence was hypoplastic in both ears (Figure 3). On the coronal view HRCT, the position of the tympanic segment of the facial nerve appeared normal, locating itself below the lateral semicircular canal (Figure 4). The mastoid segment of the facial nerve in the right ear did not appear as a single distinct fiber but was obscured. In the left ear, two separate fibers were observed (Figure 5). Normally, we would expect to see one continuous fiber.

Surgical Findings

**Figure 6:**
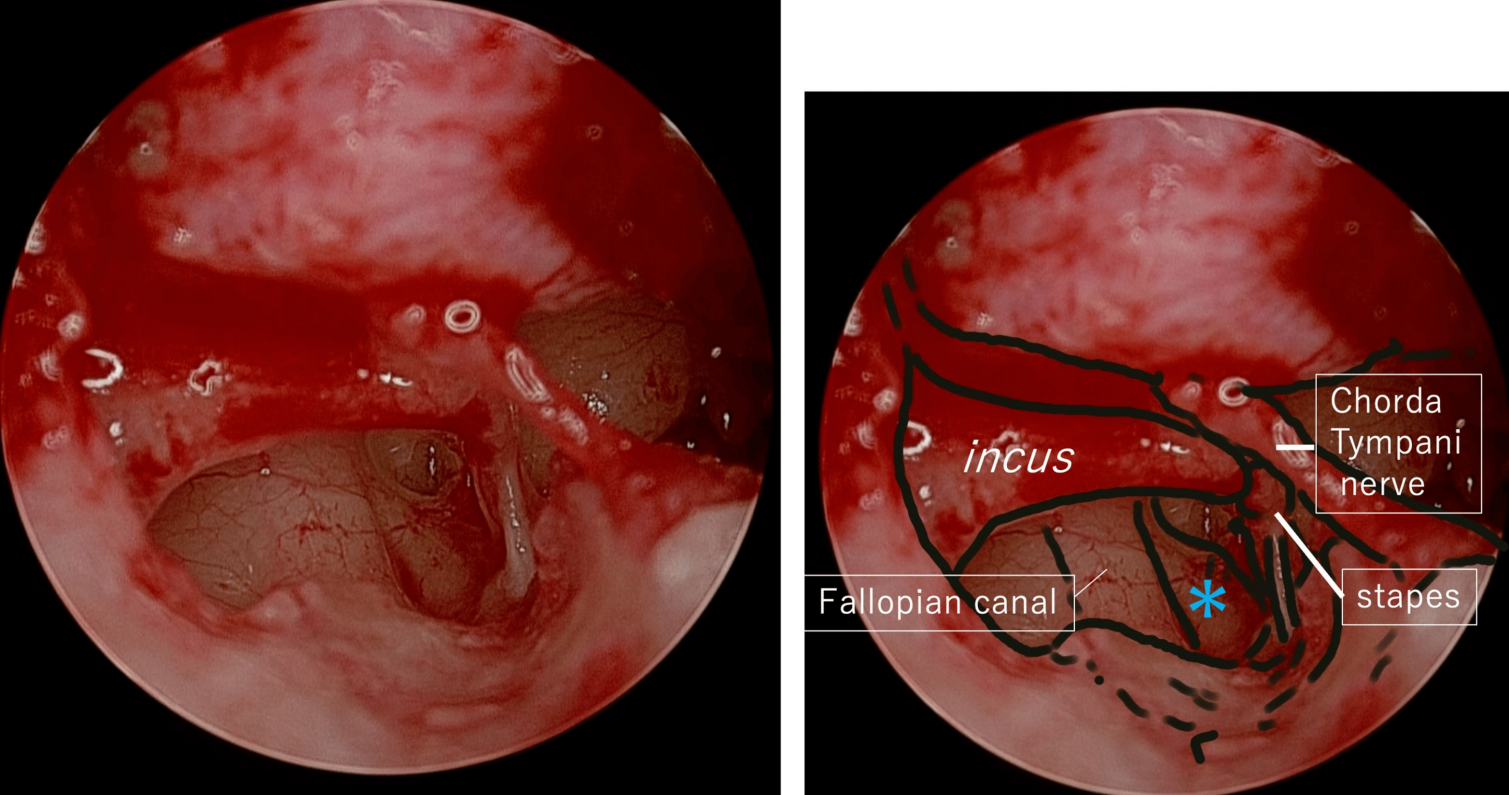
Endoscopic view of the abnormal facial nerve hanging over the footplate of stapes (Case 1). The right figure is the annotated version of the raw image on the left. The facial nerve is marked with an asterisk.

**Figure 7:**
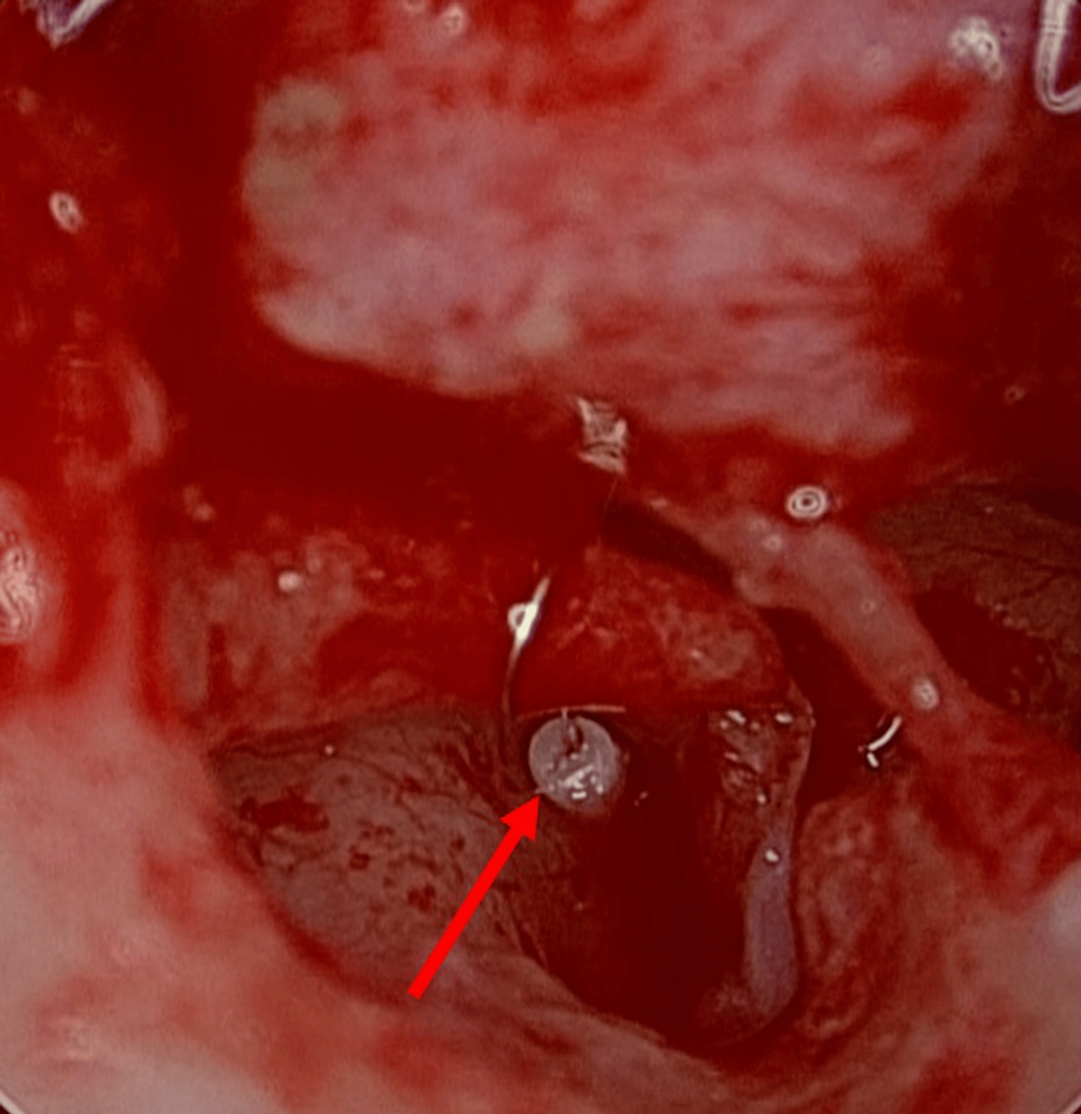
Stapedotomy results. Tefron wire piston (arrow) was inserted into the footplate of the stapes while avoiding the facial nerve (Case 1).

An operation was performed on her right ear with trans-canal endoscopic ear surgery (TEES) under general anesthesia. After raising the tympano-meatal flap, we found the ptosis of the facial nerve, confirmed by the nerve integrity monitor (NIM). It was hanging over the footplate of the stapes (Figure 6). The mobility of the stapes was extremely poor, but the malleus and incus looked normal, and the mobility was good. The confirmed diagnosis was stapes ankylosis. Stapedotomy using hand drills was performed, where the Teflon wire piston (prosthesis 0.6 mm × 4.5 mm) was inserted into the footplate of the stapes while avoiding the facial nerve, and the head of the stapes was cut with a pair of scissors (Figure 7).

**Figure 8:**
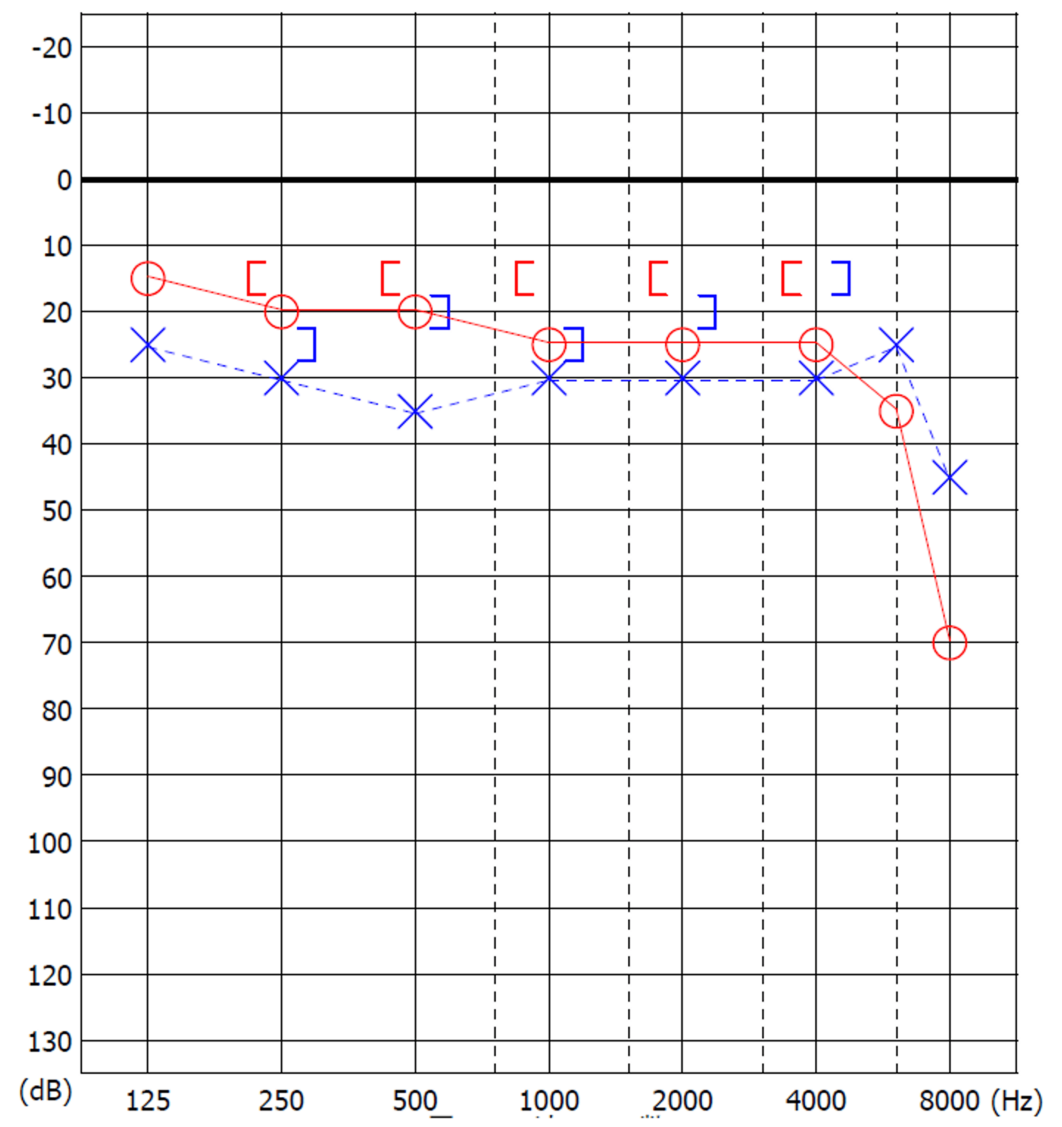
Postoperative pure tone audiogram showing improved hearing loss in right ear (Case 1). The average hearing level improved to 23.3 dB in the right ear. Right ear, circle; Left ear, cross

After the surgery, the average hearing level improved to 23.3 dB in the right ear (Figure 8). There were no complications, such as delayed facial palsy, sensorineural hearing loss, and vertigo. Six months have passed since the operation, with no particular problems, such as worsening of hearing or facial paralysis.

Case 2

**Figure 9:**
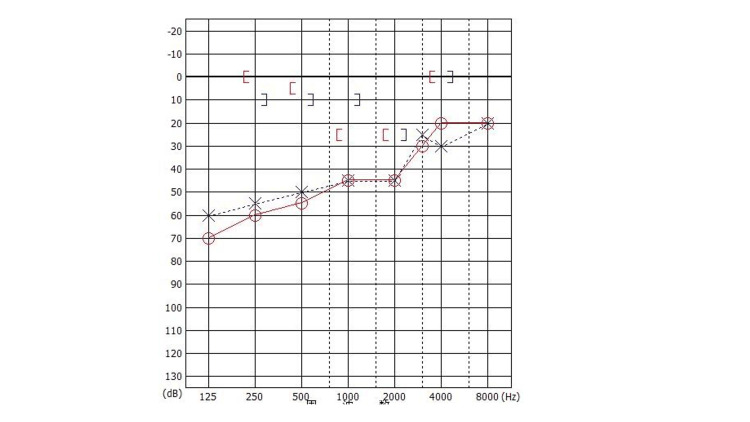
Preoperative pure tone audiogram showing mixed hearing loss in both ears (Case 2). The average hearing level was 48.3 dB for the right ear (circle) and 46.7 dB for the left ear (cross).

A 32-year-old female visited our hospital with bilateral hearing impairments from childhood. She had no history of surgery. The external ear and tympanic membrane were normal. Pure-tone audiometry showed mixed hearing loss in both ears. Her average hearing levels (500 Hz, 1 kHz, and 2 kHz) were 48.3 dB and 46.7 dB in the right and left ear, and the stapedial reflex was absent in both ears (Figure 9).

Radiological Findings

**Figure 10:**
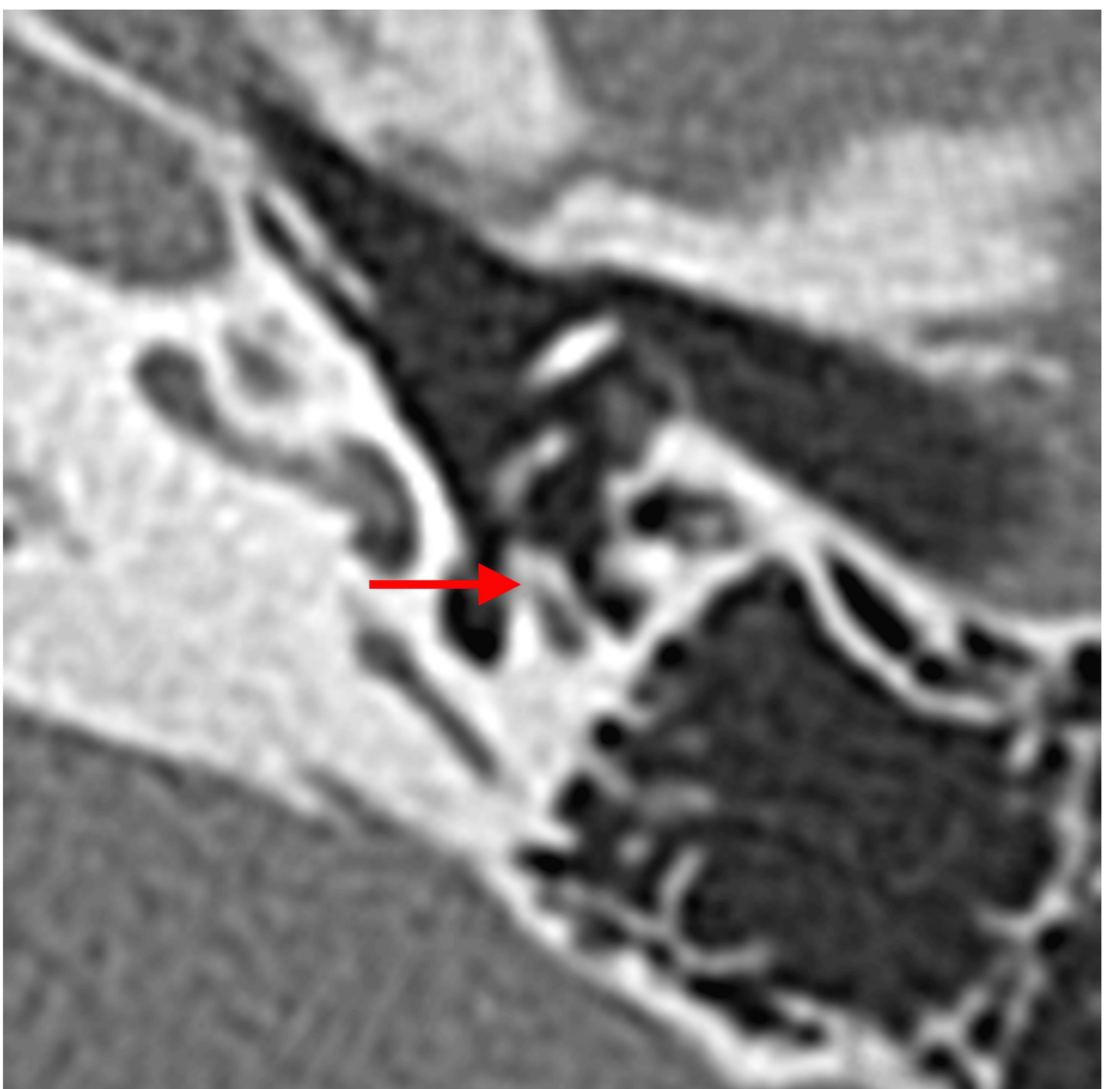
Axial HRCT image of the right temporal bone (Case 2) showing a normal pyramidal eminence (arrow). HRCT, High-resolution computed tomography

**Figure 11:**
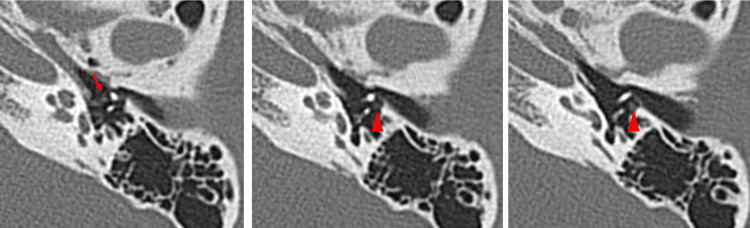
Three consecutive sections (0.5 mm thickness) of axial HRCT images of the left temporal bone (Case 2) showing an abnormal soft tissue lesion appearing at the medial side of the malleus (arrow) and at the lateral side of the stapes (arrowheads). HRCT, High-resolution computed tomography

**Figure 12:**
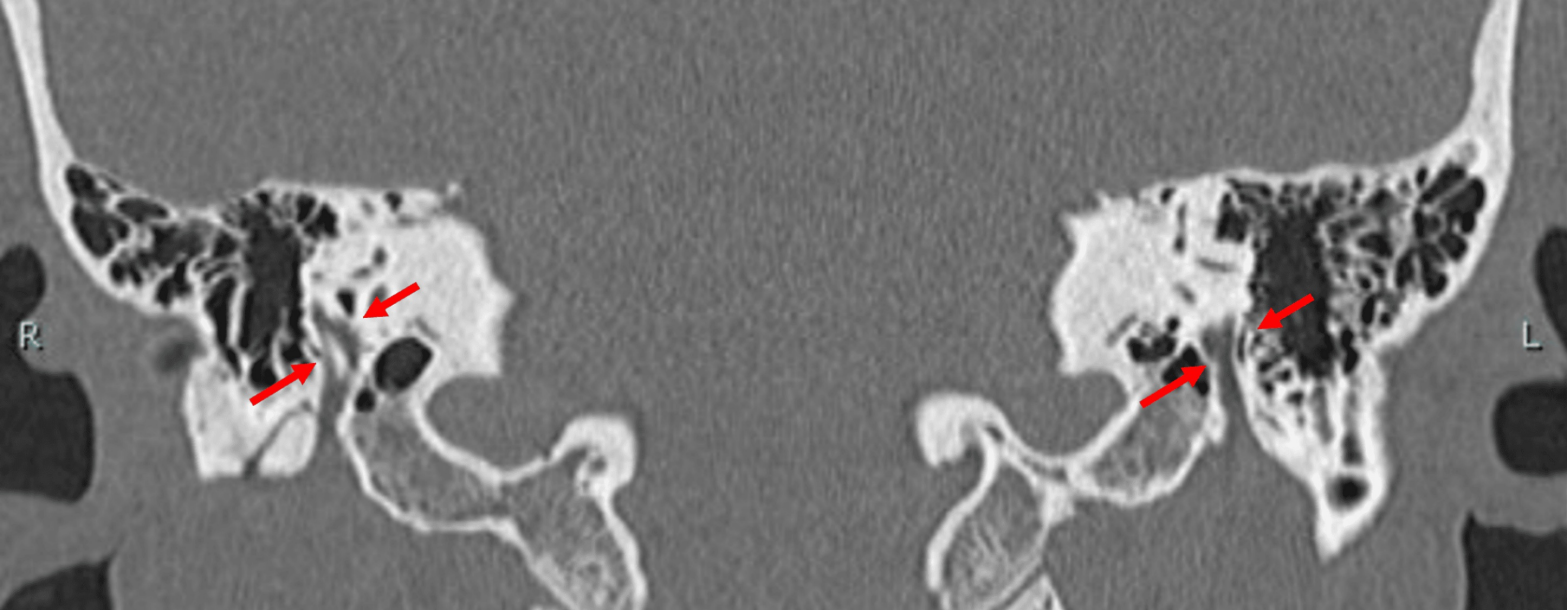
Coronal HRCT image of the temporal bone showing the mastoid segment of facial nerve appearing as branched fiber (arrows) on both (R = right and L = left) ears (Case 2). HRCT, High-resolution computed tomography

On the temporal bone HRCT (120 kVp, high-resolution matrix (512 × 512), section thickness 0.5 mm, and Field of View, or FOV, 15-20 cm), the morphology of the malleus and incus looked normal. There was no hypodense demineralized plaque in the region of the fissure ante fenestra. The pyramidal prominence appeared normal (Figure 10). There was no clear image of nerve fibers on the CT in the area where the tympanic segment is usually found. Only the abnormal lesion-like soft tissue was visible on the lateral side of the stapes and the medial side of the malleus (Figure 11). On the coronal HRCT, the mastoid portion of the facial nerve was branched in both ears (Figure 12). 

Surgical Findings

**Figure 13:**
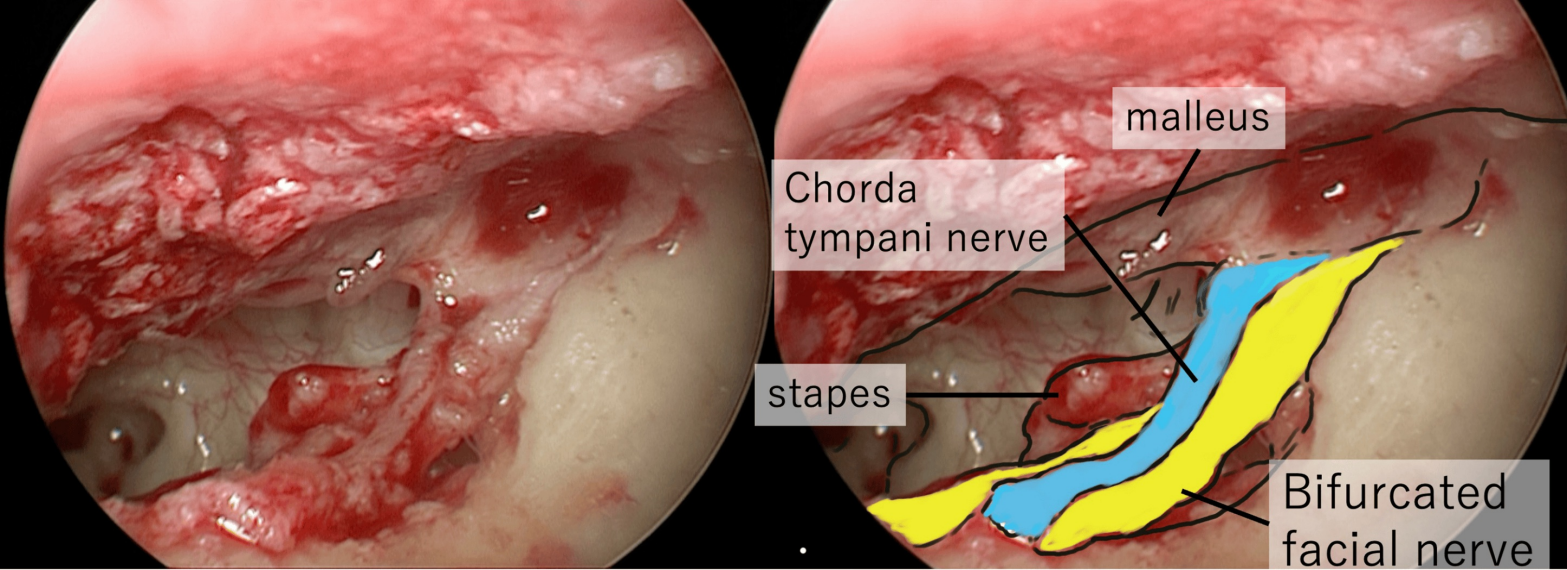
Endoscopic view of the abnormal facial nerve emerging in the shallow portion of the tympanic sulcus, running from the incudo-malleolar joint, between the incus and the chorda tympani nerve and into the temporal bone medial to the canaliculus chorda tympani. The right figure is the annotated version of the raw image on the left.

An operation was performed on her left ear with TEES under general anesthesia. During lateral tympanotomy, a facial nerve fiber emerged in the shallow portion of the tympanic sulcus, where the facial nerve does not typically appear. The fiber was running from the incudo-malleolar joint, between the incus and the chorda tympani nerve, and ran into the temporal bone medial to the canaliculus chorda tympani (Figure 13). Using an NIM, we confirmed the fiber was the facial nerve, judging from the response of the NIM to the fibers being stronger compared to the response to the chorda tympani. The mobility of the stapes was extremely poor. The mobility of the incus was also somewhat poor, even though the morphology of the malleus and the incus looked normal. We couldn’t keep enough working space to do the stapes surgery, so we discontinued the operation. We considered performing stapes surgery following posterior tympanotomy, but decided not to proceed due to the risk of facial nerve injury and the lack of a guaranteed sufficient hearing improvement.

## Discussion

When an abnormal stapes is suspected, the surgeon should suspect facial nerve abnormalities in the tympanic and mastoid segments, because they are derivatives of the second branchial arch [3,4]. The indicators for the suspicion of stapes ankylosis in our cases were the congenital onset of hearing loss, the absence of the typical finding of hypodense demineralized plaques in the region of the fissula ante fenestram, and the hypoplastic pyramidal prominence on HRCT [12]. The facial nerve anomalies in the tympanic segment were not apparently visible with preoperative HRCT for Cases 1 and 2 but were predictable based on the HRCT images of the facial nerve branch in the mastoid segment with a coronal view. The abnormalities found during surgery were facial nerve ptosis in Case 1, which is one of the frequently reported types (Table 1) [1,2,6,13], while the abnormal route in Case 2 is of a kind that has been rarely reported. The resolution and condition of the HRCT we used seem adequate for visualizing features of the course of the facial nerve, where it is surrounded by bone, such as the mastoid segment. In the coronal view of HRCT, the mastoid segment of the facial nerve can be observed as a fiber, and it was relatively easy to depict abnormalities in the course of the facial nerve. On the other hand, in cases where the nerve runs through the tympanic cavity without being enclosed by bone, differentiation from other soft tissue, such as inflammation, becomes extremely difficult. This situation highlights the importance of integrating imaging insights during preoperative assessments and using them as a critical indicator of the possibilities of facial nerve abnormalities. Other examples of preoperative detection of facial nerve abnormalities in the mastoid segment include the superficial mastoid segment, anteromedial displacement, and duplication for cochlear-implanted children [14], hypoplastic and anteriorly displaced mastoid segments in patients with microtia, and bilateral bifid intratemporal facial nerve without other associated congenital middle ear or inner ear abnormalities [7,15].

**Table 1:** Types of facial nerve abnormalities associated with middle ear malformations. Based on previous reports, we have documented the number and types of facial nerve abnormalities associated with middle ear malformations.

References	Number of ears (n)	Number of cases associated with facial nerve anomaly (n_fn_)＿ percentage (n_fn_/n x 100)	Type of facial nerve abnormalities	Number of cases associated with facial nerve anomaly type (n_fnt_)＿ percentage (n_fnt_/n_fn _x 100)
Hao et al. (2018) [1]	256	82＿32.0%	Ptosis	50＿61.0%
	-	-	Bifurcation surrounding the oval window	3＿3.7%
	-	-	Transverse promontory	3＿3.7%
Jahrsdoerfer (1981) [2]	54	13＿24.1%	Dehiscent and ptosis	4＿30.8%
	-	-	Running inferior to the oval window	4＿30.8%
	-	-	Traversing above the stapes	2＿15.4%
	-	-	Crossing the middle ear cavity	1＿7.8%
Thomeer et al. (2012) [6]	107	10＿9.3%	Ptosis	10＿100%
Senturk et al.(2020) [13]	28	11＿39.3%	Dehiscent and ptosis	11＿100%

The HRCT image of the tympanic segment appeared normal, except for soft tissue shadows around the ossicles in Case 2. Distinguishing such soft tissue shadows of facial nerve abnormalities from lesions (e.g., prior otitis media) was difficult and could not be done affirmatively. The position of the facial nerve, caudal to the lateral semicircular canal, was normal, contrary to the report [16], which suggests a tendency for an increased distance between the lateral semicircular canal and the facial nerve, as well as a decreased distance between the facial nerve and the round window in ossicular malformation cases. Utilizing the visualization capability of TEES, we could unexpectedly locate the facial nerve in the tympanic sulcus covering the incus and were able to decide that the difficulty of surgical manipulation of the stapes and the potential risk of Gusher were too great to continue with the stapes surgery. It is our view that posterior tympanotomy with a microscopic approach was not a feasible alternative either. Possible future approaches include the use of reconstructed 3D images for finer reoperation planning, which is underway [17].

## Conclusions

Two cases of congenital middle ear malformations, associated with facial nerve abnormalities, highlighted the critical role of preoperative CT imaging in detecting subtle features of facial nerve anomalies. Our findings demonstrate that identifying specific CT markers, such as nerve branching in the mastoid segment or soft tissue shadows around the ossicles, can significantly enhance surgical planning and reduce the risk of facial nerve injury. This underscores the benefits of thorough radiological evaluations in managing difficult cases.
